# The diagnostic values of circulating miRNAs for hypertension and bioinformatics analysis

**DOI:** 10.1042/BSR20180525

**Published:** 2018-08-29

**Authors:** Xiaoyi Zhang, Xiaoyan Wang, Jian Wu, Juan Peng, Xin Deng, Yi Shen, Chunjie Yang, Jie Yuan, Yunzeng Zou

**Affiliations:** 1 Department of Geriatrics, Zhongshan Hospital, Fudan University, Shanghai, China; 2 Shanghai Institute of Cardiovascular Diseases, Zhongshan Hospital and Institutes of Biomedical Science, Fudan University, Shanghai, China

**Keywords:** biomarkers, hypertension, KEGG analysis

## Abstract

Few studies have compared the performances of those reported miRNAs as biomarkers for hypertension in a same cohort, we aimed to comprehensively examine the performances of those reported miRNAs as biomarkers for hypertension and identify the genes and pathways targetted by these miRNAs. Serum samples were collected from patients hospitalized for hypertension in Zhongshan Hospital. Gene expressions of 25 miRNAs were compared between hypertension and normal groups. Receiver operating characteristic (ROC) curves were used to evaluate the accuracy of those miRNAs as biomarkers for hypertension. miRWALK2.0 and Kyoto Encyclopedia of Genes and Genomes (KEGG) enrichment analysis were performed to predict the target genes and pathways of selected miRNAs. A total of 164 participants were enrolled, amongst which 53 were patients with hypertension, 111 were normal population. *MiR-122-5p* (area under curve (AUC): 0.750), *miR-199a-3p* (AUC: 0.744), *miR-208a-3p* (AUC: 0.743), *miR-423-5p* (AUC: 0.740), and *miR-223-5p* (AUC: 0.718) showed better performance than others, and the best performance was the combination of *miR-199a-3p, miR-208a-3p, miR-122-5p*, and *miR-223-3p* (AUC: 0.80). Pathway analysis revealed that 94 pathways enriched with genes targetted by *miR-199a-3p, miR-208a-3p, miR-122-5p, miR-223-5p*. FoxO signaling was enriched with genes targetted by all the three miRNAs (*miR-199a-3p, miR-208a-3p, miR-122-5p*). The combination of *miR-199a-3p, miR-208a-3p, miR-122-5p*, and *miR-223-3p* has a good diagnostic performance for hypertension, and multitudes of possible mechanisms/pathways through which dysregulation of these miRNAs may impact risk of hypertension.

## Introduction

Hypertension is the leading global risk factor for cardiovascular diseases [1], and has been long recognized as the most common risk factor for cardiovascular disease [1,2], chronic renal failure [3], stroke [1], and so on [4]. The adverse effects and unsatisfactory control rate of hypertension calls for earlier detection and more accurate therapy [5,6].

The understandings of miRNA [7–9] have advanced the diagnosis and treatment of hypertension from proteomics [10,11] and genomics [12] to epigenetics [13]. MiRNAs not only play a role in the pathogenesis of hypertension [14], but also can be used as biomarkers for hypertension [15]. Amount of circulating miRNAs have been identified as biomarkers for hypertension in different populations [15], however, few studies have compared those miRNAs in a same cohort of hypertension patients. These studies were limited in the used variable normalization methods and their results remain controversial. Hence, we cannot get a picture of the strength of those different miRNAs as biomarkers for hypertension.

In addition, miRNA profiles associated with hypertension in humans have not yet been studied in a comprehensive way. Many of the previous publications aimed at studying single miRNAs and focussed on limited downstream effects [16,17], and very few studies looked into the systematic interactions between miRNA and gene signaling pathways.

Thus, we aimed to comprehensively examine the performance of those reported miRNAs as biomarkers for hypertension in a same cohort and identify the genes and pathways targetted by these miRNAs.

## Materials and methods

### Patients and control subjects

Serum samples were collected from patients enrolled in the China National Heart Failure Registry (CN-HF). The CN-HF is a national, multicentered, prospective, and observational registry study, led by Shanghai Institute of Cardiovascular Diseases, Zhongshan Hospital (head unit) with 50–100 secondary and tertiary hospitals involved. All subjects signed an informed written consent to participate in the study that was approved by Ethical Committee of Zhongshan Hospital, Fudan University, China, which is according to the principles stated in the Declaration of Helsinki (approval number: B2012-140(2)).

Hypertension is defined as systolic blood pressure ≥ 140 mmHg, or diastolic blood pressure ≥ 90 mmHg, or on antihypertensive medications. Normal population enrolled the subjects who have no cardiovascular disease or any other disease. The levels of brain natriuretic peptide (BNP) and cTNT were obtained from the results of biochemical test, left ventricular ejection fraction (LVEF) was obtained from echocardiography in our hospital.

### Serum preparation and RNA isolation

Blood samples (approximately 5 ml) were collected from each donor and placed in a serum separator tube. Samples were processed within 1 h. Separation of the serum was accomplished by centrifugation at 800×***g*** for 10 min at room temperature, followed by a 15-min high-speed centrifugation at 10000×***g*** at room temperature to completely remove the cell debris. The supernatant serum was recovered and stored at −80°C until analysis.

For RNA isolation, miRNA was extracted from 200 μl of each serum sample and eluted in 30 μl of RNase-free water using miRcutes serum/plasma miRNA isolation kit (Tiangen, Beijing, China) according to the manufacturer’s instructions.

### cDNA synthesis and RT-qPCR

The miRNA isolated from blood sample was polyadenylated and reverse transcribed to cDNA in a final volume of 20 μl using miRcute miRNA First-Strand cDNA Synthesis Kit (Tiangen, Beijing, China). Real-time PCR was performed in duplicate measurements using miRcute Plus miRNA qPCR Detection Kit (SYBR Green) (Tiangen, Beijing, China). The miRNA-specific primer sequences were designed by a biologics company (Tiangen, Beijing, China). Each amplification reaction was performed in a final volume of 20 μl containing 1 μl of the cDNA, 0.2 mM of each primer and 1× miRcute Plus miRNA Premix (with SYBR ROX). At the end of the PCR cycles, melting curve analyses as well as electrophoresis of the products on 3.0% agarose gels were performed in order to validate the specific generation of the expected PCR product. Each sample was run in duplicate for analysis.

### Pathway analysis

To predict the target genes for the candidate miRNAs, we used the miRWALK2.0 (http://zmf.umm.uni-heidelberg.de/apps/zmf/mirwalk2) [18,19], an open-access webserver that integrates 12 target prediction algorithms. Kyoto Encyclopedia of Genes and Genomes (KEGG) enrichment analysis was performed for overlapped genes using DAVID (https://david.ncifcrf.gov/) [20,21]. A Fisher’s exact test *P*-value <0.05 was used to identify significantly targetted pathways in KEGG and enriched gene target pathways obtained from these databases.

### Data analysis

Data were expressed in terms of mean ± S.D. or median (interquartile range) for numeric variables and as number (percent) for categorical variables. Comparisons of continuous variables amongst groups were performed by the Student’s *t* test or Mann–Whitney test, if appropriate. For comparison of categorical variable, chi-square test was used. The correlationship between miRNAs and biochemical indicators in hypertension patients was evaluated by Pearson correlation coefficient. Statistic analysis was performed with SPSS 22.0 (SPSS Inc., Chicago, Illinois, U.S.A.).

## Results

### Characteristics of the enrolled individuals

A total of 164 participants were enrolled, amongst which 53 were patients with hypertension, 111 were normal population. As was shown in Table 1, the age of hypertension patients was significantly higher than normal population (64.43 ± 9.60 compared with 46.29 ± 9.09, *P*<0.001), while the percentage of male gender has no difference (69.8 compared with 73.9%, *P*=0.581). In addition, the levels of α2-macroglobulin, β2-microglobulin, triglyceride in hypertension patients were significantly higher than normal population, while the levels of hemoglobin, glomerular filtration rate, high-density lipoprotein were significantly lower than normal population (Table 1).

**Table 1:** Characteristics of the enrolled individuals

Index	Hypertension *n*=53	Control *n*=111	*P*-value
Male gender, *n* (%)	37 (69.8%)	82 (73.9%)	0.581
SBP	132.17 ± 13.29	115.70 ± 15.50	<0.001
Age	64.43 ± 9.60	46.29 ± 9.09	<0.001
α2-macroglobulin	1.62 (1.40–2.22)	1.55 (1.23–1.76)	0.006
β2-microglobulin	1.99 (1.70–2.51)	1.62 (1.41–1.86)	<0.001
HB	133.17 ± 13.40	146.14 ± 18.38	<0.001
WBC	6.12 ± 1.41	6.44 ± 1.66	0.228
GFR	79.66 ± 20.70	91.82 ± 15.99	<0.001
TC	5.09 ± 0.83	4.41 ± 1.05	<0.001
HDL	1.22 ± 0.28	1.44 ± 0.37	0.001

Abbreviations: GFR, glomerular filtration rate; HB, hemoglobin; HDL, high-density lipoprotein; SBP, systolic blood pressure; TC, triglyceride; WBC, white blood cell.

### Selection of the reference genes for circulating miRNA

Considering that few studies evaluated the stability and superiority of serum *miR*-16, U6 snRNA (U6), 5S ribosomal RNA (5S), *miR-19b, miR-15b, miR-24, let-7i* as reference genes in cardiovascular disease, we first assess the suitability of those seven miRNAs as normalizers in cardiovascular disease using BestKeeper, NormFinder, and comparative ΔCq analysis. Our results showed that *miR-16* and let-7i have the best performance, and 5S is not suitable as reference gene. Thus, in the present study *miR*-16 was used as the reference gene [22].

### The predictive value of the selected miRNAs for hypertension

Significant negative correlations of *miR-133a-3p* (r: 0.175, *P*=0.031), *miR-199a-3p* (r: 0.293, *P*<0.001), *miR-1-3p* (r: 0.276, *P*=0.001), *miR-208a-3p* (r: 0.299, *P*<0.001), *miR-423-5p* (r: 0.299, *P*<0.001), *miR-223-3p* (r: 0.337, *P*<0.001), *miR-122-5p* (r: 0.368, *P*<0.001) were observed with hypertension. (Table 2 and Figure 1A). In contrast, statistically increased levels of *miR-18b-5p* (r: −0.259, *P*=0.001), *miR-20b-5p* (r: −0.306, *P*=0.001), *miR-548c-3p* (r: −0.231, *P*=0.007), *miR-499a-5p* (r: −0.244, *P*=0.005) were observed in hypertension patients (Table 2 and Figure 1B). The comparative Δ*C*_t_ levels of miRNAs between normal group and hypertension group were shown in Supplementary Table S1.

**Figue 1:**
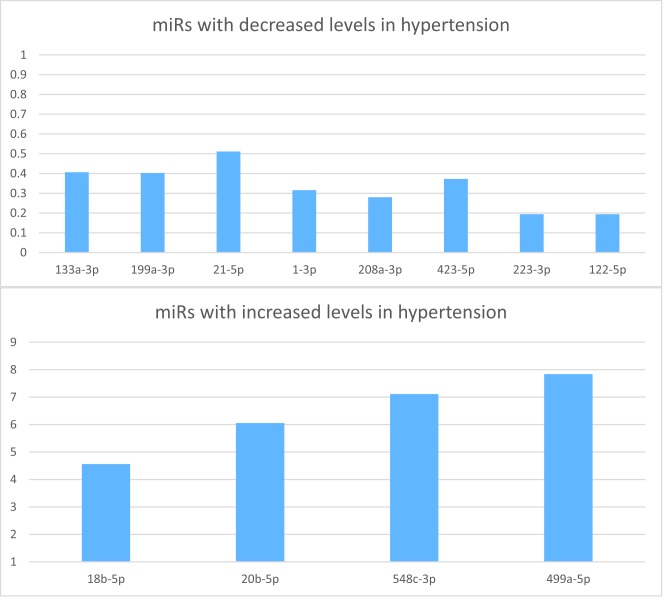
2
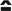
ΔΔ*C*_t_ of the selected miRNAs

**Table 2:** The correlation of levels of miRNAs with hypertension

MiRNA	RQ	Pearson correlation	*P*-value
1-5p	0.582	0.132	0.094
133a-3p	0.406	0.175	0.031
23a-5p	0.544	0.140	0.124
199a-3p	0.403	0.293	<0.001
20b-3p	1.964	−0.136	0.111
155-5p	0.559	0.141	0.084
195-5p	0.716	0.094	0.231
21-5p	0.511	0.193	0.013
320a	0.782	0.073	0.376
1-3p	0.316	0.276	0.001
208a-3p	0.280	0.299	<0.001
423-5p	0.372	0.299	<0.001
106b-5p	0.940	0.015	0.852
21-3p	0.706	0.090	0.291
23a-3p	0.542	0.153	0.053
126-5p	0.578	0.138	0.107
133b	0.595	0.096	0.235
675-3p	2.035	−0.193	0.020
223-3p	0.194	0.337	<0.001
Let-7i-5p	0.768	0.066	0.479
208b	1.024	−0.005	0.952
19b-3p	1.253	−0.064	0.952
122-5p	0.194	0.368	<0.001
18b-5p	4.562	−0.259	0.001
20b-5p	6.057	−0.306	0.001
548c-3p	7.109	−0.231	0.007
499a-5p	7.838	−0.244	0.005

**Figure 2:**
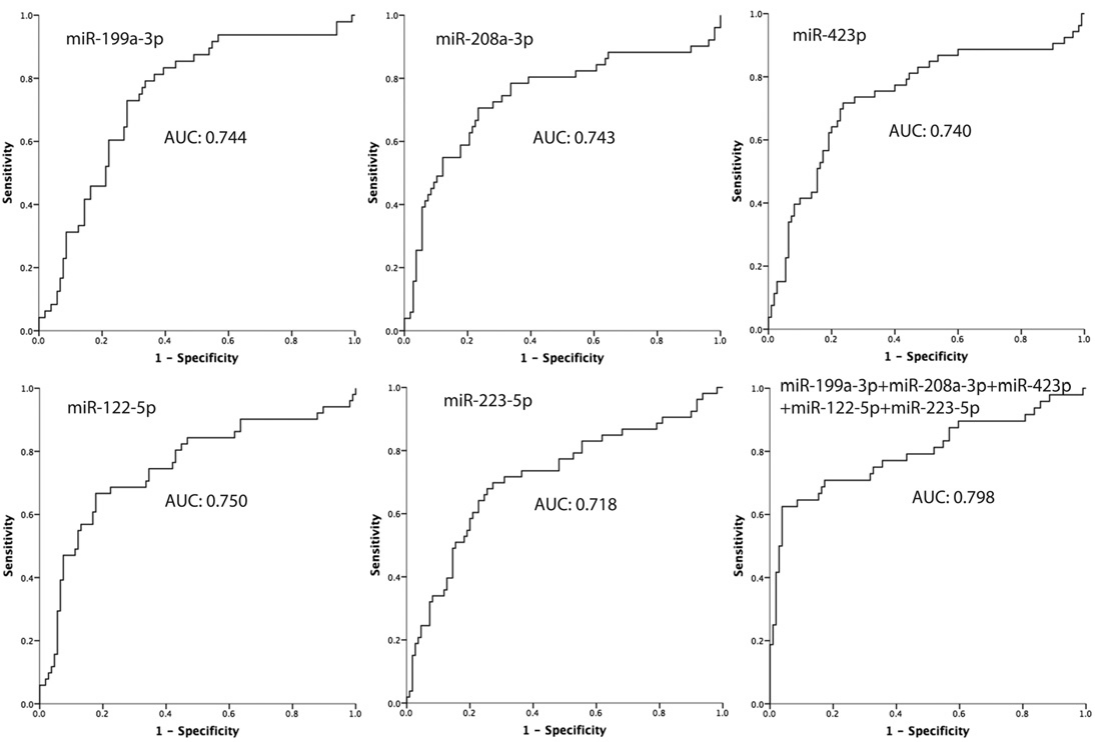
ROC curve for the first four miRNAs which have a good prediction ability for hypertension

After identification of those miRNAs that were differentially expressed in a discovery phase, we further evaluated the diagnostic performances of those selected miRNAs by receiver operating characteristic (ROC) analysis. *MiR-122-5p* (area under curve (AUC): 0.750), *miR-199a-3p* (AUC: 0.744), *miR-208a-3p* (AUC: 0.743), *miR-423-5p* (AUC: 0.740), *miR-223-5p* (AUC: 0.718) showed better performance than others (Figure 2).

**Figure 3:**
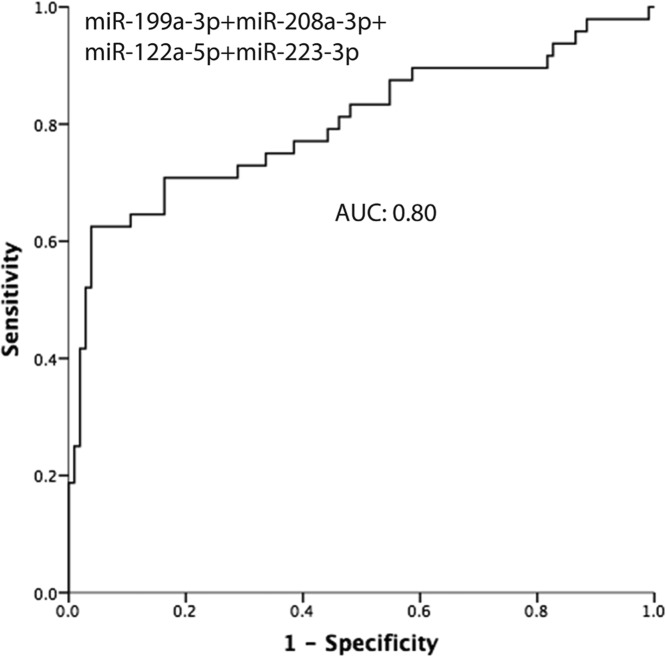
The predictive value of the combination of *miR-199a-3p, miR-208a-3p, miR-122-5p*, and *miR-223-3p*

A combined analysis using those five miRNAs were used to explore the value of the combination, and the AUC was 0.798 (Figure 2). We also calculated the AUC of the combined analysis using the four from the five miRNAs, and the best performance was the combination of *miR-199a-3p, miR-208a-3p, miR-122-5p*, and *miR-223-3p* (AUC: 0.80) (Figure 3).

### Correlation of the selected miRNAs with the biochemical indexes

Several miRNAs not only showed significant correlation with hypertension, but also had high association with indicators reflecting myocardial injury and cardiac function. For myocardial injury, ten of the selected miRNAs were highly related with cTNT, five of which were significantly associated with both hypertension and myocardial injury (*miR-1-3p* (r: 0.238, *P*:0.003), *miR-208a-3p* (r: −0.169, *P*:0.033), *miR-20b-5p* (r: −0.328, *P*<0.001), *miR-548c-3p* (r: −0.175, *P*=0.042), *miR-499a-5p* (r: −0.208, *P*=0.016). For cardiac function, *miR-20b-5p* was also related with BNP (r: −0.266, *P*=0.003) and LVEF (r: 0.219, *P*=0.015) (Table 3).

**Table 3:** Correlation of the selected miRNAs with the biochemical indexes

Indicator	miRNA	Pearson correlation	*P*-value
BNP	*miR-1-3p*	0.231	0.004
	*miR-20b-5p*	−0.266	0.003
	*miR-548c-3p*	−0.207	0.016
	*miR-499a-5p*	−0.261	0.002
cTNT	*miR-1-3p*	0.238	0.003
	*miR-208a-3p*	−0.169	0.033
	*miR-20b-5p*	−0.328	<0.001
	*miR-548c-3p*	−0.175	0.042
	*miR-499a-5p*	−0.208	0.016
LVEF	*miR-208a-3p*	−0.212	0.007
	*miR-20b-5p*	0.219	0.015
	*miR-548c-3p*	0.189	0.028

### Targetted genes and pathways for the four candidate miRNAs

**Figure 4:**
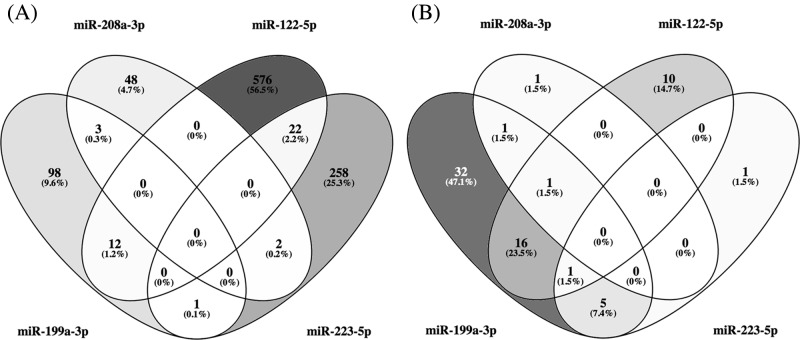
GO and KEGG analysis for the four candidate miRNAs (**A**) Venn’s diagram of genes targetted by each of the four candidate miRNAs. (**B**) Venn’s diagram of pathways enriched with genes targetted by the four candidate miRNAs with *P*<0.05. Pathways related to cancer were eliminated.

Using miRWALK2.0 software, 1060 predicted genes were identified to be targetted by the four candidate miRNAs. The number of genes targetted by each of the miRNAs are shown in boxes in Figure 4A. Since many of the genes were targetted by multiple miRNAs, the total of unique genes was only 1020. The multiple color Venn diagram presented in Figure 4A showed the distribution of these genes according to the candidate miRNAs that targetted them.

To identify pathways enriched with these genes, a KEGG pathway analysis was performed. This analysis identified 94 pathways enriched with genes targetted by *miR-199a-3p, miR-208a-3p, miR-122-5p*, and *miR-223-5p*. Many of the pathways, however, overlapped, so in total there were 68 unique pathways enriched at statistical significance of *P*<0.05.

Figure 4B shows distribution of the enriched pathways as a Venn diagram according to the candidate miRNAs, and Table 3 provides the name of the pathways with information about degree of enrichment with targetted genes. There was no pathway enriched with genes targetted by all four miRNAs with statistical significance. Pathways of FoxO signaling were enriched with genes targetted by three miRNAs (*miR-199a-3p, miR-208a-3p, miR-122-5p*). Twenty five pathways were enriched with genes that were targetted by two miRNAs (15 pathways when excluded the cancer pathways). The remaining 45 pathways were enriched with genes targetted only by one miRNA. We further analyzed the pathways enriched with genes targetted by the combination of four miRNAs, the most statistically significant of which were focal adhesion (*P*<3.0 × 10^−6^), glioma (*P*<1.0 × 10^−5^), endocytosis (*P*<2.0 × 10^−4^), MAPK signaling pathway (*P*<4 × 10^−4^) (Table 4).

**Table 4:** KEGG pathways enriched by genes targetted by the four candidate miRNAs (*P*<0.05)

		*miR-199a-3p*		*miR-208a-3p*		*miR-122-5p*		*miR-223-5p*	
miRNA(s)	KEGG pathways	Target genes	*P*-value	Target genes	*P*-value	Target genes	*P*-value	Target genes	*P*-value
All four miRNAs	NA	NA	NA	NA	NA	NA	NA	NA	NA
*miR-199a-3p, miR-122-5p, miR-223-5p*	Glioma	5	0.0014	—	—	7	0.017	5	0.014
*miR-199a-3p, miR-208a-3p, miR-122-5p*	FoxO signaling pathway	8	<6.0 × 10^−5^	4	0.016	10	0.027	—	—
*miR-199a-3p, miR-208a-3p*	Epstein–Barr virus infection	7	0.003	4	0.04	—	—	—	—
*miR-199a-3p, miR-223-5p*	Focal adhesion	14	<3.0 × 10^−6^	—	—	—	—	8	0.03
	Pathways in cancer	15	<8.0 × 10^−7^					12	0.009
	Melanoma	7	<2.0 × 10^−5^					6	0.004
	Prostate cancer	5	0.004					6	0.010
	Endocytosis	6	0.04					8	0.020
*miR-199a-3p, miR-122-5p*	Proteoglycans in cancer	12	2.0 × 10^−9^	—	—	14	0.01	—	—
	Renal cell carcinoma	6	1.2 × 10^−4^			8	0.004		
	Pancreatic cancer	6	1.2 × 10^−4^			9	0.001		
	Sphingolipid signaling pathway	7	2.6 × 10^−4^			10	0.01		
	Osteoclast differentiation	7	4.2 × 10^−4^			12	0.003		
	Central carbon metabolism in cancer	5	<0.002			7	0.02		
	Toxoplasmosis	6	<0.002			9	0.03		
	Choline metabolism in cancer	5	0.07			8	0.04		
	Insulin resistance	5	0.009			10	0.007		
	Colorectal cancer	4	0.01			8	0.004		
	Neurotrophin signaling pathway	5	0.01			9	0.04		
	Epithelial cell signaling in *Helicobacter pylori* infection	4	0.01			7	0.02		
	Pertussis	4	0.01			7	0.03		
	Insulin signaling pathway	5	0.02			11	0.01		
	Hepatitis B	5	0.02			10	0.04		
	Tuberculosis	5	0.04			15	0.002		

## Discussion

Our study comprehensively examined the performance of those previously reported miRNAs in a same population. Our study showed that the combination of 199a-3p, 208a-3p, 122-5p, and 223-3p (AUC: 0.80) has a satisfactory diagnostic performance. Besides, some of those miRNAs also have correlation with myocardial injury (*miR-1-3p, miR-208a-3p, miR-20b-5p, miR-548c-3p, miR-499a-5p*), cardiac function (*miR-20b-5p*). Bioinformatics analysis showed that these four candidate miRNAs target the expression of more than 1020 genes that may impact 68 KEGG pathways. Although no pathway enriched with genes targetted by all four miRNAs with a statistical significance, pathways of FoxO signaling was enriched with genes targetted by three miRNAs (*miR-199a-3p, miR-208a-3p*, and *miR-122-5p*, respectively). Overall, our study identified four circulating miRNAs which target thousands of genes and many dozens of pathways. The interpretation and experimental validation of our findings creates a formidable challenge considering that our study measured circulating miRNAs and not intracellular in humans. Furthermore, not all human miRNAs are present in animals. Following this, we discuss our findings in the context of the limited literature regarding the pathways and miRNAs involved.

FoxO signaling pathway contains 132 genes/proteins, and in our study, 10 of these genes were predicted to be targetted by *miR-122-5p*, eight and four of these genes targetted by *miR-199a-3p* and *miR-208a-3p*, respectively. This pathway regulates the expression of genes in cellular physiological events including apoptosis, cell-cycle control, glucose metabolism, oxidative stress resistance, and longevity. Especially, FoxO signaling pathway was reported to play an important role in both vascular smooth muscle cells and endothelial cells, mediating the process of cell proliferation [23], vascular homeostasis [24], and age-related vascular changes [25]. Savai et al. [26] reported that FoxO1 in pulmonary artery smooth muscle cells (PASMCs) are a critical integrator of multiple signaling pathways driving pulmonary hypertension (PH), and reconstitution of FoxO1 activity offers a potential therapeutic option for PH. In addition, expression of p-FoxO/FoxO was elevated in both in spontaneously hypertensive rat (SHR), indicating the important role of FoxO signaling pathway in hypertension.

Except for FoxO signaling pathway, there were 15 pathways enriched with genes that were targetted by two miRNAs (excluding the cancer pathway). Epstein–Barr virus infection pathways targetted by both *miR-199a-3p* and *miR-208a-3p*, have been reported to be associated with secondary pulmonary arterial hypertension [27], while the mechanism remains to be discovered. Focal adhesion pathway plays an essential role in important biological processes including cell motility, cell proliferation, cell differentiation, regulation of gene expression, and cell survival. And total focal adhesion kinase was proved to be increased in renovascular hypertension, indicating the role of focal adhesion pathway in hypertension [28]. Endocytosis is a mechanism for cells to remove ligands, nutrients, and plasma membrane (PM) proteins, and lipids from the cell surface, bringing them into the cell interior and was associated with portal hypertension [29].

For pathways targetted by both *miR-199a-3p, miR-122-5p*, sphingolipid signaling pathway (targetted by) is known to have second messenger functions in a variety of cellular signaling pathways, and recently sphingosine-1-phosphate receptor 1 signaling was proved to regulate blood flow and pressure [30]. Insulin resistance and insulin signaling pathway have been suggested to be associated with hypertension in both clinical (metabolic syndrome) and basic trials. However, little has been done to identify the association of osteoclast differentiation and neurotrophin signaling pathway with hypertension. There were also three ways participating in infection, such as toxoplasmosis [31], pertussis [32], epithelial cell signaling in *Helicobacter pylori* infection [33], which have been proved to be related with hypertension, while pathways involving hepatitis B and tuberculosis have not been proved to be associated with hypertension. However, mechanisms of those pathways interacted with hypertension remains to be investigated.

In contrast with the results of our pathway analysis, which showed that those circulating miRNAs might target a large number of genes in multiple pathways, many of the previous publications aimed on studying single miRNAs and focussed on limited downstream effects. For example, studies on *miR-223* have focussed its impact on the regulation of specific downstream gene target. *miR-223* was down-regulated in human PAH lungs, in both the right heart and lungs from rodent models of PH. Down-regulation of *miR-223* triggers PARP-1 and insulin-like growth factor 1 receptor (IGF-1R) overexpression, subsequent pathologic DNA damage repair, and increased proliferation [16,17]. The relationship between miRNAs and target genes *in vivo* is a complex network. Thus far, very few studies looked into the systematic interactions between miRNA and gene signaling pathways. In the present study, we performed pathway enrichment analysis and have identified top canonical pathways highly relevant to the pathogenesis of hypertension, including the FoxO signaling pathway.

Our study showed that in hypertension patients, the levels of *miR-320a* [34], *miR-199a-3p* [35,36] were lower than control group, while the levels of *miR-20b-3p/5p* [37], *miR-208b* [38], *miR-499a-5p* [38] increased, which kept the same with previous studies, indicating that the expression levels of these miRNAs were stable. However, the trend of many selected miRNAs in hypertension remains controversial in different studies, such as *miR-1-3p/5p* [39,40], *miR-23a-3p/5p* [34,35], *miR-155-5p* [41–43], *miR-195-5p* [34,43,44], *miR-133a-3p* [36,39], *miR-223-3p* [16,45], *miR-122-5p* [43,46]. In addition, the expression level of *miR-423-5p* and *miR-106b-5p* in our study was contrary to the previous reports [37]. There are several reasons that can explain these inconsistencies. First, the population in the previous studies include hypertension of different types (such as gestational hypertension, PH, renal vascular hypertension) or hypertension combined with different complication, which can effect the levels of hypertension. Second, miRNAs have many pathways to participate in the pathophysiology of hypertension, and other factors may affect the levels of hypertension (such as dietary structure, coffee consumption, exercise, pathogenesis, and so on). Those results suggested that the pathogenesis and complication of hypertension and the pathways of miRNAs should be taken into consideration when we identify miRNA as biomarkers for hypertension.

Our study found the best four biomarkers for hypertension (*miR-122-5p, miR-199a-3p, 208a-3p, miR-423-5p*, and *miR-223-5p*), and identified the best combination of those miRNAs (199a-3p, 208a-3p, 122-5p, and 223-3p), giving more information on miRNAs as biomarkers for hypertension. Furthermore, four of those miRNAs (*miR-1-3p, miR-20b-5p, miR-548c-3p, miR-499a-5p*) were significantly correlated with cTNT, indicating that those miRNAs maybe used as indicators for myocardial infarction. Those five miRNAs have also been shown to be correlated with myocardial infarction in previous studies [47]. In addition, *miR-20b-5p* was also related with BNP (relative risk (RR): −0.266, *P*=0.003) and LVEF (RR: 0.219, *P*=0.015), indicating the association between *miR-20b-5p* and cardiac function. In consistence with our results, *miR-20b-5p* had been proved to be significantly increased in response to hypertension-induced heart failure, and correlated with the levels of BNP.

The limitations of our study are: (i) because of amounts of miRNAs have been reported to be related with hypertension, we can only select those important, representative miRNAs instead of detecting them one by one. (ii) Considering that the the expressing levels of miRNAs may be different in different types of hypertension, we did not carry out a subgroup analysis according to the types of hypertension.

## Conclusion

The combination of 199a-3p, 208a-3p, 122-5p, and 223-3p has a good diagnostic performance for hypertension, and multitudes of possible mechanisms/pathways through which dysregulation of these miRNAs may impact risk of hypertension.

## Clinical perspectives

- Few studies have comprehensively examined the performances of those reported miRNAs as biomarkers for hypertension and identify the genes and pathways targetted by these miRNAs.
- The combination of 199a-3p, 208a-3p, 122-5p, and 223-3p has a good diagnostic performance for hypertension, and multitudes of possible mechanisms/pathways through which dysregulation of these miRNAs may impact risk of hypertension.
- Our results give some information on the systematic interactions between miRNA and gene signaling pathways and provide some clues for mechanism research on miRNA and hypertension.

## Supporting information

**Supplemental Table 1:** Levels of delta ct of microRNAs between normal group and hypertension group

## Abbreviations

AUC: area under curve
BNP: brain natriuretic peptide
CN-HF: China National Heart Failure Registry
Cq: quantification cycle
Ct: threshold cycle
cTNT: cardiac Troponin T
FoxO: The forkhead box O
KEGG: Kyoto Encyclopedia of Genes and Genomes
LVEF: left ventricular ejection fraction
MAPK: mitogen activated protein kinase
PAH: pulmonary arterial hypertension
PH: pulmonary hypertension
PARP-1: poly(ADP-ribose) polymerase 1
ROC: receiver operating characteristic
RR: relative risk
RT-qPCR: quantitative reverse transcription polymerase chain reaction
5S: 5S ribosomal RNA
