# Supplementary material for: The diagnostic values of circulating miRNAs for hypertension and bioinformatics analysis
Source: Biosci Rep. 2018 Aug 29;38(4):BSR20180525. doi: 10.1042/BSR20180525 (PMC6147777; doi:10.1042/BSR20180525)
Supplement: Supplementary file 1 [file bsr20180525_Supp1.pdf]

Supplemental Table 1. Levels of delta ct of microRNAs between normal group and hypertension group

MicroRNAs	Delta ct of microRNAs		P-value
	Normal group	Hypertension group	
miR-1-5p	0.00±2.65	0.78±2.90	0.949
miR-133a-3p	0.00±3.15	1.30±3.84	0.142
miR-23a-5p	-0.34 (-1.59-1.53)	1.44 (-0.45-2.65)	0.009
miR-199a-3p	-0.15 (-0.87-0.79)	1.02 (0.45-2.41)	<0.001
miR-20b-3p	0.00±-0.97	3.14±3.56	0.777
miR-155b-5p	-0.15 (-1.34-1.15)	1.01 (-0.05-1.66)	0.005
miR-195-5p	-0.24 (-0.80-0.59)	0.15 (-0.44-0.95)	0.062
miR-21-5p	-0.22 (-0.77-0.68)	0.86 (-0.013-1.562)	<0.001
miR-320a	0.38 (-1.01-1.26)	0.51 (-0.67-1.38)	0.206
miR-1-3p	0.00±2.37	1.66±3.34	0.100
miR-208a-3p	0.00±2.43	1.84±3.36	0.375
miR-423-5p	-0.04 (-1.05-1.07)	1.68 (0.53-2.67)	<0.001
miR-106b-5p	-0.77 (-0.97-0.70)	-0.85 (-1.47-0.00)	0.024
miR-21-3p	0.00±2.31	0.50±2.93	0.242
miR-23a-3p	0.18 (-1.35-1.77)	1.15 (0.28-1.85)	0.006
miR-126-5p	-0.20 (-0.98-1.01)	0.85 (-0.06-1.40)	0.001
miR-133b	0.00±4.04	0.75±2.42	0.001
miR-675-3p	0.12 (-1.05-1.12)	-1.14 (-2.43- -0.21)	0.001
miR-223-3p	-0.15 (-1.59-1.13)	-1.11 (-2.81—0.53)	<0.001
Let-7i-5p	0.13 (-1.68-1.28)	1.47 (-1.17-2.25)	0.104
miR-208b	0.00±2.91	-0.03±3.10	0.588
miR-19b-3p	-0.20 (-1.06-1.12)	-0.89 (-1.66 -0.089)	0.052
miR-122-5p	-0.04 (-1.44-1.31)	2.61 (0.57-3.79)	<0.001
miR-18b-5p	0.00±3.97	-2.19±3.65	0.235
miR-20b-5p	0.69 (-1.45-2.35)	-1.02 (-3.64-0.03)	<0.001
miR-548c-3p	0.00±6.10	-2.83±4.93	0.061
miR-499a-5p	0.00±5.59	-2.97±5.69	0.803
